# The Accuracy of Artificial Intelligence in the Endoscopic Diagnosis of Early Gastric Cancer: Pooled Analysis Study

**DOI:** 10.2196/27694

**Published:** 2022-05-16

**Authors:** Pei-Chin Chen, Yun-Ru Lu, Yi-No Kang, Chun-Chao Chang

**Affiliations:** 1 Department of Internal Medicine Taipei Medical University Hospital Taipei Taiwan; 2 Department of General Medicine Taipei Medical University Hospital Taipei Taiwan; 3 Department of Anesthesiology Wan Fang Hospital Taipei Medical University Taipei Taiwan; 4 Evidence-Based Medicine Center Wan Fang Hospital, Taipei Medical University Taipei Taiwan; 5 Institute of Health Behaviors and Community Sciences College of Public Health National Taiwan University Taipei Taiwan; 6 Cochrane Taiwan Taipei Medical University Taipei Taiwan; 7 Department of Health Care Management College of Health Technology National Taipei University of Nursing and Health Sciences Taipei Taiwan; 8 Division of Gastroenterology and Hepatology Department of Internal Medicine Taipei Medical University Hospital Taipei Taiwan

**Keywords:** artificial intelligence, early gastric cancer, endoscopy

## Abstract

**Background:**

Artificial intelligence (AI) for gastric cancer diagnosis has been discussed in recent years. The role of AI in early gastric cancer is more important than in advanced gastric cancer since early gastric cancer is not easily identified in clinical practice. However, to our knowledge, past syntheses appear to have limited focus on the populations with early gastric cancer.

**Objective:**

The purpose of this study is to evaluate the diagnostic accuracy of AI in the diagnosis of early gastric cancer from endoscopic images.

**Methods:**

We conducted a systematic review from database inception to June 2020 of all studies assessing the performance of AI in the endoscopic diagnosis of early gastric cancer. Studies not concerning early gastric cancer were excluded. The outcome of interest was the diagnostic accuracy (comprising sensitivity, specificity, and accuracy) of AI systems. Study quality was assessed on the basis of the revised Quality Assessment of Diagnostic Accuracy Studies. Meta-analysis was primarily based on a bivariate mixed-effects model. A summary receiver operating curve and a hierarchical summary receiver operating curve were constructed, and the area under the curve was computed.

**Results:**

We analyzed 12 retrospective case control studies (n=11,685) in which AI identified early gastric cancer from endoscopic images. The pooled sensitivity and specificity of AI for early gastric cancer diagnosis were 0.86 (95% CI 0.75-0.92) and 0.90 (95% CI 0.84-0.93), respectively. The area under the curve was 0.94. Sensitivity analysis of studies using support vector machines and narrow-band imaging demonstrated more consistent results.

**Conclusions:**

For early gastric cancer, to our knowledge, this was the first synthesis study on the use of endoscopic images in AI in diagnosis. AI may support the diagnosis of early gastric cancer. However, the collocation of imaging techniques and optimal algorithms remain unclear. Competing models of AI for the diagnosis of early gastric cancer are worthy of future investigation.

**Trial Registration:**

PROSPERO CRD42020193223; https://www.crd.york.ac.uk/prospero/display_record.php?RecordID=193223

## Introduction

Gastric cancer is the fifth most common cancer and the third leading cause of cancer deaths worldwide, contributing to 19.1 million disability-adjusted life years in 2017 [1,2]. Its primary risk factors are *Helicobacter pylori* infection and a family history of gastric cancer [3,4]. Despite advancements in endoscopic, surgical, and systemic therapies, the global 5-year survival rate of those with gastric cancer remains low (25%-30%) [5]. Gastric cancer has an excellent prognosis at early stages, with a 5-year survival rate of approximately 95%, but it has a median survival rate of less than one year at advanced stages [6,7]. Its favorable early prognosis is reflected in the lower mortality rates of gastric cancer in East Asia, which can be ascribed to the implementation of nationwide screening [8]. This reinforces the importance of early diagnosis. However, gastrointestinal endoscopy, the standard detection method for early gastric cancer, has an unsatisfactory sensitivity of 70% and is operator dependent [9]. Despite efforts to increase the detection rate, a valid screening method has yet to be developed [10,11]. The recent advancement in artificial intelligence (AI) systems, which provides highly accurate and efficient image recognition, may indicate a solution to this problem.

Although significant increases in AI exist in many fields and in health care [12-19], AI has various definitions [20]. According to the cognitive modeling approach, AI can be seen as machines that perform or exhibit actions corresponding to intelligence such as human behavior [20,21]. Machine learning, a subset of AI, involves studying how computers learn to improve task performance through experience without being programmed. This learning is achieved through various approaches. For instance, support vector machines, widely used in data classification, are machine learning algorithms that work by calculating the best separating plane for distinguishing between different objects. Deep learning, another machine learning method, simulates the multiple hierarchical layers of neural networks to make decisions based on features extracted from massive training data. Convolutional neural networks are deep learning algorithms primarily used in image recognition [22].

Since the breakthrough of deep learning in the 2010s, the use of AI in clinical practice has increased dramatically [22,23], and many studies have applied AI for screening or diagnosis [24-27]. Several studies have provided promising results for the AI-assisted endoscopic diagnosis of gastric cancer [28]. In a multicenter case control study of 84,424 participants, a deep learning–aided system demonstrated a detection rate of upper gastrointestinal cancer comparable to that of an expert endoscopist [29]. Other studies have investigated the diagnostic accuracy of AI for gastric polyps and the invasion depth of gastric cancers [30,31]. Nevertheless, the rate of detection of early gastric cancer, which allows for prompt intervention and increased survival rates, remains low. Multiple studies on the AI-assisted diagnosis of early gastric cancer have been conducted in the past 5 years, but results have been inconsistent and highly variable. Furthermore, the role of AI in early gastric cancer is more important than in advanced gastric cancer since early gastric cancer is not easily identified in clinical practice; however, to our knowledge, past syntheses appear to have limited focus on the population with early gastric cancer. Thus, we investigated the performance of AI-assisted endoscopic diagnosis of early gastric cancer.

## Methods

### Definition

Early gastric cancer was defined as mucosal and submucosal (T1) gastric cancer irrespective of lymph node involvement. Studies involving advanced gastric cancer, precancerous lesions such as intestinal metaplasia and dysplasia, and gastric cancer without specific annotations were excluded. The accuracy of AI was defined as the area under the hierarchical summary receiver operating characteristic curve or the area under the curve (AUC).

### Study Search and Selection Strategy

This meta-analysis was performed according to the PRISMA (Preferred Reporting Items for Systematic Reviews and Meta-Analyses) guidelines. We systematically searched the PubMed, Embase, Cochrane Library, and Web of Science databases for studies that assessed the diagnostic accuracy of AI in early gastric cancer from endoscopic images from database inception to June 2020. We used “gastric cancer,” “endoscopy,” and “artificial intelligence” as relevant terms with Boolean operators “OR” and “AND” (Multimedia Appendix 1). Two authors, P-CC and L-YR, independently screened the study titles and abstracts. Studies that used AI to diagnose early gastric cancer from endoscopic images were included. Studies that did not provide a 2×2 contingency table were not included in the final analysis. This study was registered in PROSPERO (registration CRD42020193223).

### Study Quality Assessment and Data Extraction

The quality of the included studies was assessed independently by 2 authors (P-CC and L-YR) on the basis of the revised Quality Assessment of Diagnostic Accuracy Studies (QUADAS-2), and all disagreement was resolved through discussion with the third author (Y-NK). The assessment included risk of bias and applicability to the QUADAS-2 domains: patient selection, index test, reference standard, and flow and timing. From the included studies, we extracted data on the number of endoscopic images of lesions diagnosed as early gastric cancer (ie, true positive), the number of endoscopic images of benign lesions misdiagnosed as malignant (ie, false positive), the number of endoscopic images of malignant lesions misdiagnosed as benign (ie, false negative), and the number of endoscopic images of benign lesions correctly diagnosed as benign (ie, true negative). We also extracted data on the country of origin, AI methods, and image modalities used.

### Study Outcomes and Statistical Analysis

The primary outcome was the accuracy of AI to diagnose early gastric cancer from endoscopic images. Secondary outcomes focused on the sensitivity analysis of (a) different AI methods, (b) endoscopic imaging modalities, (c) studies that compared AI and endoscopist performance, (d) studies that evaluated larger gastric lesions (>20 mm), (e) studies that simply differentiated abnormal and normal lesions rather than using pathological staging, and (f) studies that separated the training and testing data sets during AI training. Sensitivity analysis was conducted if a subgroup contained more than two studies. We only assessed the heterogeneity of the included studies. Following extraction, the data were primarily analyzed using STATA 14 (StataCorp LP, StataCorp) except for subgroups with fewer than four studies. The midas and metandi commands were used to determine sensitivity, specificity, and AUC and analyze the summary receiver operating characteristic (SROC) and hierarchical summary receiver operating characteristic (HSROC) curves. Basic formulas for the analyses were as follows:

ln DOR = (logit TPR) - (logit FPR) (1)

proxy for the threshold = (logit TPR) - (logit FPR) (2)

TPR of SROC =1/[1/(1+ea/(1-b))× (FPR/(1-FPR))(1+b)/(1-b)] (3)

In the formulas, “a” is the intercept, “b” is slope, and DOR refers to the diagnostic odds ratio. Moreover, TPR is the true positive rate, and FPR is the false-positive rate. The modchk tool was used to examine goodness-of-fit and bivariate normality before SROC analysis in a bivariate mixed-effects model. The metabias command and the pubbias syntax were used to perform the Egger test and Deeks funnel plot asymmetry tests, respectively. The Egger test for diagnostic meta-analysis was based on the formula proposed by Hasselblad and Hedges, and the formula is mainly to detect publication bias detection via testing standard normal deviate among the included studies [32,33].

standard normal deviate = a + b × SE_(d)^-1^_ (4)

In the regression model, with intercept “*a*” and slope “*b*,” the standard normal deviation could be estimated by using diagnostic *d* divided by SE of the diagnostic *d*. The metaprop package in STATA was mainly used to synthesize the sensitivity and specificity. *I^2^* statistics were used to determine levels of heterogeneity via the formula as follows:

I2 = ((Q − df)/Q) × 100 (5)

where Q refers to Cochran Q, and *df* is the degree of freedom. Because R software (The R Foundation) does not restrict the number of observations used in the meta-analysis, it was used for sensitivity analysis if subgroups consisted of fewer than four studies. Indeed, a meta-analysis in R could be carried out when more than two studies report the same outcome by pooling data with logit transformation and Clopper-Pearson interval method (also called exact binomial interval) based on inverse variance. Function metaprop in package meta for R was applied to carry out sensitivity analysis, and the mada package in R was used to calculate the pooled accuracy. Besides, the metagen package in R was used to synthesize endoscopist performance because of the lack of detailed data on each endoscopist.

## Results

### Literature Search and Review

**Figure 1:**
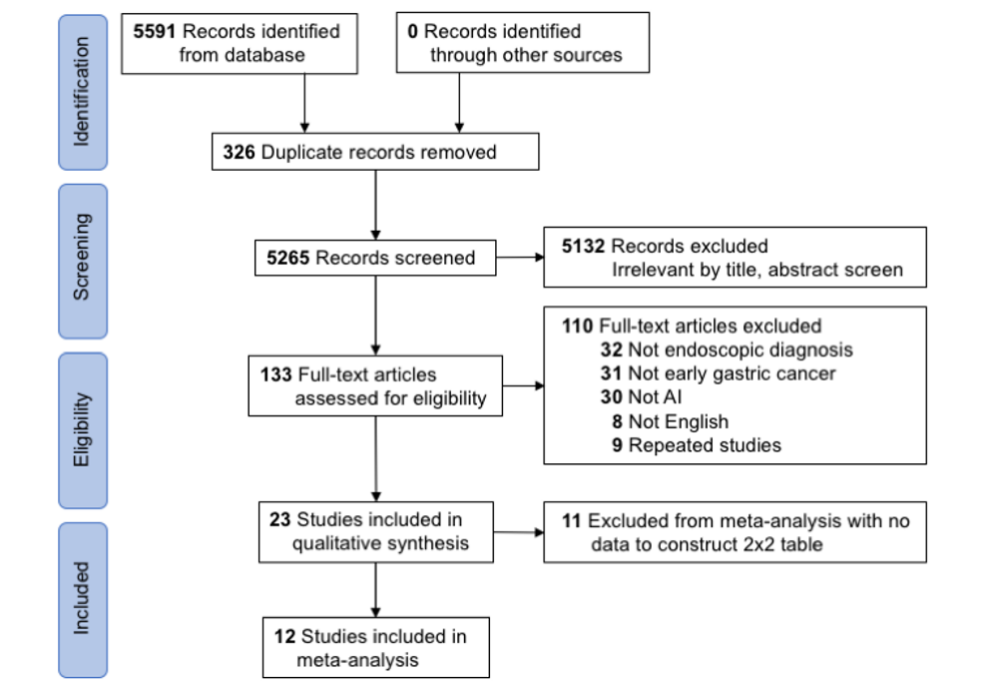
Flowchart of the study selection process according to the PRISMA (Preferred Reporting Items for Systematic Reviews and Meta-Analyses) format. AI: artificial intelligence.

Of the 5591 studies identified in the literature review, 5265 underwent title and abstract screening after duplication removal. The flowchart of the literature review process was constructed according to the PRISMA flowchart format (Figure 1). We excluded 5132 irrelevant studies and assessed the eligibility of the remaining 133 studies through full-text reading. Studies evaluating nonearly gastric cancer (eg, advanced gastric cancer and metaplasia) were excluded. Overall, 23 studies investigated the performance of AI on early gastric cancer diagnosis from endoscopic images. Finally, 12 studies comprising a total of 11,685 cases were included in the meta-analysis [34-45].

### Study Description and Bias Assessment

Detailed information on the 12 studies is listed in Table 1. All studies were conducted in Asia, including Japan (k=8), China (k=2), and Korea (k=2), in or after 2012. All were case control studies with testing data sets containing 81 to 3390 images. Patients in 10 studies had pathological proof of early gastric cancer, whereas in the other 2 studies, the endoscopic images were collected through description. White light imaging (WLI), narrow-band imaging (NBI), flexible spectral imaging color enhancement, and mixed imaging modalities were used in 4 (33%), 2 (17), 1 (8%), and 2 (17%) studies, respectively. Moreover, 8 (67%) studies used deep learning methods (eg, convolutional neural networks) as their AI backbone, and 3 (25%) studies employed nondeep learning methods (support vector machines and discriminant analysis of principal components). Comparisons of the diagnostic performance of AI and endoscopists were conducted in 3 (25%) studies, and 2 (17%) studies included endoscopic images of small lesions (<20 mm) in early gastric cancer. In 3 (25%) studies, the training and testing data sets were not separated for AI training. Table 1 presents a detailed description of the 12 studies.

We also assessed the quality of the studies along with the risk of bias according to the revised QUADAS-2 tool (Multimedia Appendix 2). All studies, including the 3 that failed to separate the training and testing data sets, had high bias risks for patient selection because of their retrospective design. Moreover, 2 (17%) studies assessed early gastric cancer but did not mention pathological staging. Thus, they were classified as having a high risk of bias for the index test.

**Table 1:** Characteristics of the included studies.

Study ID	Country of origin	Testing image number	Reference standard	Image modality	AI^a^ method	AI training and testing data set	Standard reference	Endoscopist comparison	Other information
Kubota et al, 2012 [43]	Japan	902	Pathology	Not mentioned^b^	Multilayer neural network	Not separated	Unclear	No	Detected with pathological grading prediction
Miyaki et al, 2013 [44]	Japan	92	Pathology	FICE^c^	SVM^d^ (scale-invariant feature transform)	Separated	Pathology	No	Differentiated early gastric cancer from noncancerous tissues
Liu et al, 2016 [41]	China	400	Pathology	Not mentioned^b^	Principal component discriminant analysis (YCbCr color space)	Separated	Pathology	No	Differentiated early gastric cancer from normal tissues
Kanesaka et al, 2018 [37]	Japan	81	Pathology	NBI^e^	SVM (grey-level co-occurrence feature)	Separated	Pathology	No	Included only depressed type early gastric cancers that were <10 mm in size
Sakai et al, 2018 [36]	Japan	926	Pathology	WLI^f^	CNN^g^ (GoogLeNet)	Not separated	Pathology	No	—^h^
Yamakawa et al, 2018 [45]	Japan	817	Unclear^i^	Not mentioned^j^	Not mentioned	Separated	Unclear	No	Differentiated early gastric cancer from nonneoplastic tissues
Cho et al, 2019 [35]	Korea	200	Pathology	WLI	CNN (Inception-Resnet-v2)	Separated	Pathology	Yes	Detected early gastric cancer with pathological grading prediction
Namikawa et al, 2019 [34]	Japan	1479^j^	Unclear^i^	WLI, NBI, Chromo^k^	CNN	Separated	Pathology	No	Differentiated early gastric cancer from gastric ulcers
Wu et al, 2019 [39]	China	200	Pathology	WLI, NBI, BLI^l^	CNN (VGG16 + Resnet-50)	Separated	Pathology	Yes	Differentiated early gastric cancer from gastritis and normal tissues
Yoon et al 2019 [42]	Korea	3390	Pathology	WLI	CNN (VGG16)	Not separated	Pathology	No	—
Horiuchi et al, 2020 [38]	Japan	258	Pathology	NBI	CNN (GoogLeNet)	Separated	Pathology	No	Differentiated early gastric cancer from *Helicobacter* *pylori*–related gastritis
Ikenoyama et al, 2020 [40]	Japan	2940	Pathology	WLI	CNN (Single-shot multiBox Detector)	Separated	Pathology	Yes	Included only early gastric lesions that were <20 mm

^a^AI: artificial intelligence.

^b^Studies that failed to mention imaging modalities.

^c^FICE: flexible spectral imaging color enhancement.

^d^SVM: support vector machine.

^e^NBI: narrow-band imaging.

^f^WLI: white light imaging.

^g^CNN: convolutional neural network.

^h^Not available.

^i^Studies that mentioned early gastric cancer but without reference to pathological staging.

^j^Studies were reported in meeting abstracts.

^k^Chromo: chromoendoscopy.

^l^BLI: blue laser imaging.

### Diagnostic Performance of AI for Early Gastric Cancer

**Figure 2:**
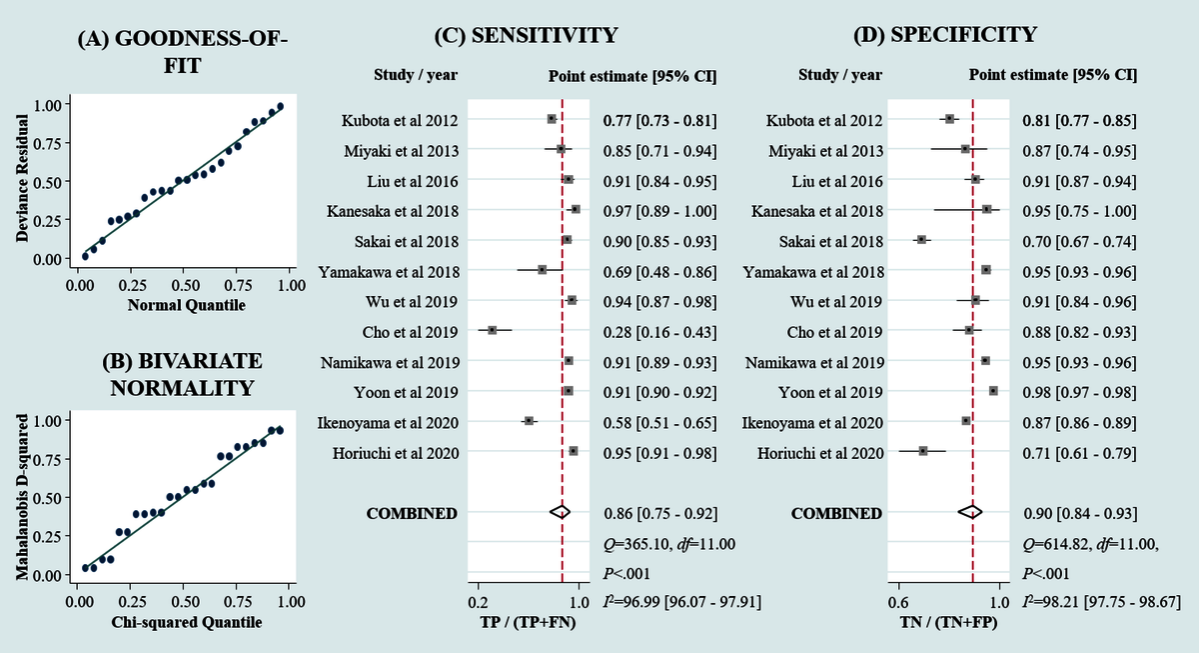
Overall sensitivity and specificity of artificial intelligence–assisted diagnosis of early gastric cancer. (A) Goodness-of-fit; (B) bivariate normality; (C) forest plot of overall sensitivity; and (D) forest plot of overall specificity. FP: false positive; TN: true negative.

**Figure 3:**
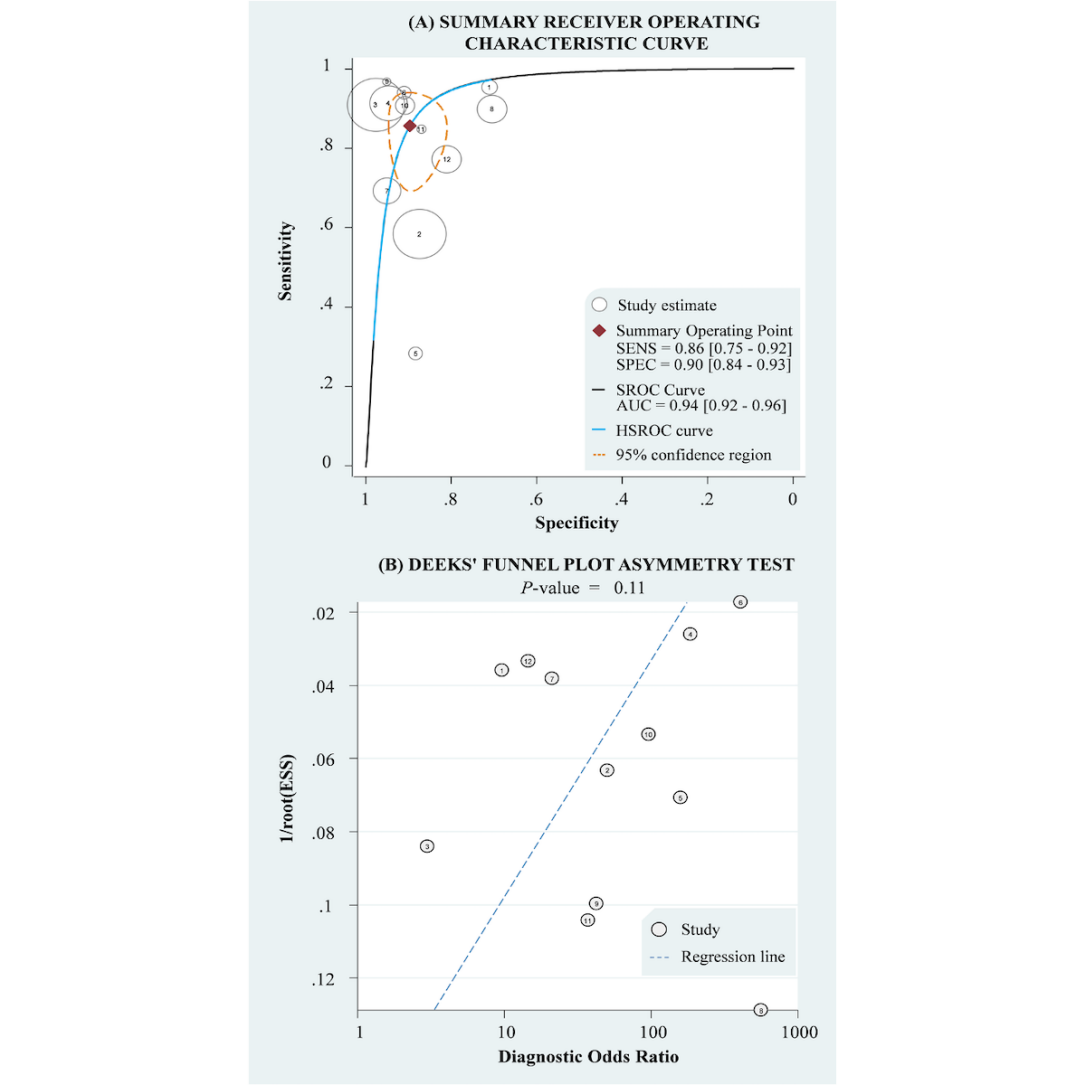
Summary receiver operating characteristic curve, HSROC, AUC, and the Deeks funnel plot asymmetry test of artificial intelligence–assisted diagnosis of early gastric cancer. AUC: area under the curve; ESS: effective sample sizes; HSROC: hierarchical summary receiver operating characteristic; SENS: sensitivity; SPEC: specificity; SROC: summary receiver operator characteristic.

To assess the diagnostic ability of AI to detect early gastric cancer from endoscopic images, we performed a meta-analysis on the selected 12 studies. Goodness-of-fit (Figure 2A) and bivariate normality (Figure 2B) demonstrated that the included data were appropriate for further analysis. The pooled sensitivity and specificity of AI were 0.86 (95% CI 0.75-0.92) and 0.90 (95% CI 0.84-0.93), respectively (Figures 2C and 2D). Empirical Bayesian predictions were consistent with the observed sensitivity and specificity (Multimedia Appendix 3). Highly heterogeneous estimates (*I*^2^>90%) necessitated subgroup analysis and sensitivity analysis. Laminated figures of the SROC and HSROC plots indicate an AUC of 0.94 (95% CI 0.92-0.96) with a confidence region (Figure 3A). However, the scatter matrix (Multimedia Appendix 4) suggests that in clinical practice, diagnosis of early gastric cancer may not substantially benefit from AI assistance. The Deeks funnel plot asymmetry test (Figure 3B) and Egger test (Multimedia Appendix 5) did not detect significant publication bias in the pooled results of AI-assisted diagnosis of early gastric cancer.

We assessed the diagnostic performance of various AI methods and endoscopic imaging modalities for early gastric cancer (Table 2). The pooled sensitivity and specificity in studies using deep learning methods were 0.84 (95% CI 0.69-0.93) and 0.88 (95% CI 0.80-0.93), respectively. Studies using nondeep learning methods had a pooled sensitivity and specificity of 0.91 (95% CI 0.86-0.95) and 0.90 (95% CI 0.87-0.93), respectively. The accuracy of the nondeep learning group (AUC=0.96) was higher than that of the deep learning group (AUC=0.93; Multimedia Appendices 6 and 7).

For endoscopic imaging modalities, studies using WLI had a sensitivity and specificity of 0.73 (95% CI 0.42-0.91) and 0.89 (95% CI 0.76-0.96), respectively. Studies using NBI reported a sensitivity and specificity of 0.96 (95% CI 0.92-0.98) and 0.83 (95% CI 0.54-0.95), respectively. The accuracy of the NBI group (AUC=0.96) was higher than that of the WLI group (AUC=0.90; Multimedia Appendices 8 and 9). Table S1 (Multimedia Appendix 10) shows a comparison of the diagnostic performance of AI and endoscopists for early gastric cancer from the three studies (n=91).

### Additional Analysis

We excluded some studies with a high risk of bias and performed sensitivity analysis on the remaining studies (Tables S2-S5 Multimedia Appendices 11-14). Furthermore, we also examined how the results were affected by studies with unknown AI methods. Sensitivity analyses indicated that pooled estimates were not seriously affected by the factors (Table 2). Lower heterogeneity and specificity were observed in endoscopist performance when we excluded studies that only evaluated small lesions and studies that predicted pathological staging (Tables S2 and S3 in Multimedia Appendices 11 and 12). Lower heterogeneity was also noted in WLI subgroups if the training and testing data sets were separated for AI training (Table S4 in Multimedia Appendix 13). No other additional analyses provided credible evidence.

**Table 2:** Pooled sensitivity, specificity, and accuracy of the studies included in the meta-analysis and sensitivity analysis.

Group (studies and number of patients)			Sensitivity (95% CI)		*I*^2^, %		Specificity (95% CI)		*I*^2^, %		AUC^a^
Overall (12 studies, n=11,685)			0.86 (0.75-0.92)		97		0.90 (0.84-0.93)		97		0.94
**Subgroup analysis on different AI^b^ methods**											
	Deep learning (8 studies, n=10,295)	0.84 (0.69-0.93)		98		0.88 (0.80-0.93)		98		0.93	
	Nondeep learning (3 studies, n=573)	0.91 (0.86-0.95)		18		0.90 (0.87-0.93)		0		0.96	
**Subgroup analysis on various imaging modalities**											
	WLI^c^ (4 studies, n=7456)	0.73 (0.42-0.91)		99		0.89 (0.76-0.96)		99		0.902	
	NBI^d^ (2 studies, n=339)	0.96 (0.92-0.98)		0		0.83 (0.54-0.95)		51		0.959	
**Sensitivity analysis**											
	Excluding studies with unknown method (11 studies, n=10,868)	0.87 (0.76-0.93)		97		0.89 (0.83-0.93)		97		0.936	
	Excluding studies with sample size <100 (10 studies, n=11,512)	0.84 (0.71-0.92)		97		0.89 (0.83-0.94)		98		0.932	
	Excluding studies without separation of testing data (9 studies, n=6467)	0.85 (0.70-0.93)		96		0.90 (0.86-0.93)		91		0.934	
	Excluding studies with any situation abovementioned (6 studies, n=5477)	0.84 (0.62-0.94)		98		0.89 (0.83-0.93)		92		0.923	

^a^AUC: area under the curve.

^b^AI: artificial intelligence.

^c^WLI: white light imaging.

^d^NBI: narrow-band imaging.

## Discussion

### Principal Findings

To our knowledge, this was the first systematic review and meta-analysis of AI-assisted endoscopic diagnosis of early gastric cancer. The accuracy, sensitivity, and specificity were 0.94, 0.86, and 0.90, respectively. High heterogeneity was noted. Sensitivity analysis revealed less heterogeneity in studies using nondeep learning AI methods and endoscopic NBI.

Our results indicate good sensitivity and specificity of AI-assisted detection of early gastric cancer. However, high heterogeneity was also noted among the included studies, which may be attributed to between-study differences in machine learning methods and imaging modalities [46]. In a meta-analysis of AI prediction of colonic polyp histology, AI performance was better when deep learning was used as a backbone and when NBI was used to identify the lesions [46]. In this study, we also investigated the roles of various machine learning methods and imaging modalities. Unfortunately, only 2 studies in the deep learning subgroup used the same deep learning algorithm, and no two studies in the nondeep learning subgroup classified the lesions according to the same features. Only 6 studies specified their endoscopic imaging modalities. Less heterogeneity was observed in the nondeep learning and NBI groups, possibly because of the compliance of early gastric cancer diagnosis to the vessel plus surface classification system under NBI. This indicates that nondeep learning methods and NBI may provide more consistent results and can be applied in clinical practice earlier than deep learning methods and WLI. Further investigations are warranted.

We assessed the diagnostic performance of AI and endoscopists (n=91) for early gastric cancer detection, which was compared in 3 studies. The endoscopists were assigned to only 1 subgroup because of the inconsistent definitions of expert and nonexpert endoscopists between studies. The sensitivity and specificity of AI were 0.67 and 0.87, respectively, and those of the endoscopists were 0.68 and 0.92, respectively. In both groups, diagnostic performance varied widely with high heterogeneity. The diagnostic performance of AI was better than that of WLI compared with other studies; a meta-analysis reported a pooled sensitivity and specificity of 48% and 67% between endoscopists and WLI, whereas those between endoscopists and NBI were 83% and 97%, respectively [47]. In this study, AI and endoscopist performance were comparable in individual studies, but this effect diminished when studies were pooled. Further research comparing AI and endoscopist performance for early gastric cancer diagnosis is required.

Only 2 of the included studies evaluated only small lesions [37,40]. Smaller lesions and mucosal lesions were less accurately detected by AI [42]. Kanesaka et al [37] included only depressed and small (<10 mm) lesions, and the AI system of nondeep learning methods was trained using a small data set of 126 images from NBI. In another study, early gastric cancer lesions less than 20 mm in diameter were included in the WLI testing data set, and the deep learning AI system was trained using a data set of 13,584 images of early and advanced gastric cancer [40]. Because these 2 studies used distinct materials and methods, their findings may not be representative. The accuracy of AI-assisted detection of small gastric cancer lesions warrants further investigation.

Some studies have explored the application of AI to other aspects of gastroendoscopy. For example, Wu et al [39] used AI to monitor endoscopic blind spots and identify regions indicative of early gastric cancer. A randomized controlled trial in China reported that AI reduced the rate of endoscopic blind spots [48]. Other studies have tested the accuracy of AI in predicting the invasion depth of gastric cancer—conventionally assessed through endoscopic ultrasound—from endoscopic images. In their study of AI-assisted simultaneous detection of gastric cancer and invasion depth, Yoon et al [42] reported a sensitivity and specificity of invasion depth of 79.2% and 77.8%, respectively. In a study by Zhu et al [31], the predicted sensitivity and specificity from the T1 to the T4 stage were 76% and 96%, respectively. Nevertheless, relevant evidence is limited, and further investigation is required.

The considerable advancement of AI in precise image recognition challenges the roles of physicians in disease diagnosis. AI systems offer certain advantages over physician diagnosis, the foremost of which are faster image processing rates and continuous work. In all included studies that specified image processing time, that of AI systems was shorter than that of endoscopists. AI assistance may reduce the risk of human error that arises from performing numerous endoscopic examinations. Moreover, the training of AI systems is considerably faster and less complicated than that of endoscopists. Well-trained AI systems learn from analyzing numerous images, whereas endoscopists rely on their individual skills and clinical experience. Training endoscopists is expensive and time-consuming because of the steep learning curve for the various image-enhancing techniques. In addition, AI may work as a double-check system during or after endoscopy, given its high sensitivity and specificity. AI allows for a second opinion, which is particularly valuable now that gastroendoscopy has been popularized and nationwide screening for gastric cancer has been implemented.

### Limitations

Our study had several limitations. First, all the included studies were retrospective case control studies performed in Asia, some of which compared early gastric cancer and normal gastric tissues, and some compared benign gastric lesions such as ulcers and gastritis. The possibility of selection bias cannot be ruled out. A randomized controlled trial comparing the diagnostic performance of AI and endoscopists for early and advanced gastric cancer (NCT04040374) is currently underway. Second, all the studies identified gastric lesions from still, clear, endoscopic images; images with blood or mucus were excluded. In daily practice, however, gastroendoscopy is recorded in video format, and still images are only captured for suspicious lesions. Blood, food debris, mucus, and foam, which reduce the accuracy of AI, are commonly encountered during examination [39]. Several studies have reported excellent accuracy of AI systems in recognizing gastric cancer from endoscopic video [39,49]. However, further studies and faster image processing rates are necessary. Third, our pooled estimates were highly heterogeneous, and the subgroup and sensitivity analyses did not substantially reduce heterogeneity. The statistical heterogeneity may be ascribed to differences in the AI methods and endoscopic imaging techniques. These potential sources of heterogeneity should be discussed in future research. At present, AI may assist endoscopists in double-checking suspicious lesions.

### Conclusions

To our knowledge, this is the first meta-analysis of the performance of AI in detecting early gastric cancer using endoscopic images. The available evidence suggests that AI can support the diagnosis of early gastric cancer; however, the collocation of imaging techniques and optimal algorithm remains unclear. Larger prospective cohort studies should be conducted to further validate the diagnostic accuracy of AI. Moreover, competing models of AI for the detection of early gastric cancer are worthy of future investigation.

## Appendix

Multimedia Appendix 1 Supplementary File 1. Search strategy (primary search strategy).

Multimedia Appendix 2 Supplementary File 2. Study quality assessment according to the QUADAS-2 (Quality Assessment of Diagnostic Accuracy Studies [revised]).

Multimedia Appendix 3 Supplementary File 3. Forest plot of empirical Bayes predicted and observed findings.

Multimedia Appendix 4 Supplementary File 4. Scatter matrix.

Multimedia Appendix 5 Supplementary File 5. Egger’s test.

Multimedia Appendix 6 Supplementary File 6. Subgroup analysis for studies that used deep learning.

Multimedia Appendix 7 Supplementary File 7. Subgroup analysis for studies without deep learning.

Multimedia Appendix 8 Supplementary File 8. Subgroup analysis for studies that used white light image.

Multimedia Appendix 9 Supplementary File 9. Subgroup analysis for studies that used narrow band imaging techniques.

Multimedia Appendix 10 Supplementary Table 1. Characteristics of the studies that compared diagnostic performance of artificial intelligence to endoscopists and its sensitivity analysis.

Multimedia Appendix 11 Supplementary Table 2. Sensitivity analysis of the studies that included gastric lesions other than small gastric cancer lesions.

Multimedia Appendix 12 Supplementary Table 3. Sensitivity analysis of the studies that do not detect early gastric cancer lesions based on pathological grading.

Multimedia Appendix 13 Supplementary Table 4. Sensitivity analysis of the studies that separated training and testing data set during artificial intelligence training.

Multimedia Appendix 14 Supplementary Table 5. Sensitivity analysis of the studies with low risk on index test.

## Abbreviations

AI: artificial intelligence
AUC: area under the curve
HSROC: hierarchical summary receiver operating characteristic
NBI: narrow-band imaging
PRISMA: Preferred Reporting Items for Systematic Reviews and Meta-Analyses
QUADAS-2: Quality Assessment of Diagnostic Accuracy Studies (revised)
SROC: summary receiver operating characteristic
WLI: white light imaging
