# Supplementary material for: The Accuracy of Artificial Intelligence in the Endoscopic Diagnosis of Early Gastric Cancer: Pooled Analysis Study
Source: J Med Internet Res. 2022 May 16;24(5):e27694. doi: 10.2196/27694 (PMC9152716; doi:10.2196/27694)
Supplement: Multimedia Appendix 1 [file jmir_v24i5e27694_app1.pdf]

Supplementary File 1
Search strategy (Primary search strategy)

Primary search strategy

#1. gastric cancer

#2. endoscopy

#3. endoscopic

#4. panendoscopy

#5. esophagogastroduodenoscopy

#6. #2 OR #3 OR #4 OR #5

#7. artificial intelligence

#8. machine learning

#9. deep learning

#10. computer-assisted

#11. computer-aided

#12. convolutional neural network

#13. predictive model

#14. prediction model

#15. reinforcement learning

#16. supervised learning

#17. unsupervised learning

#18. AI

#19. #7 OR #8 OR #9 OR #10 OR #11 OR #12 OR #13 OR #14 OR #15 OR #16 OR #17

#20. #1 AND #6 AND #19

No filter for study type, language, publication date, or age
