# Supplementary figures and images for: The Accuracy of Artificial Intelligence in the Endoscopic Diagnosis of Early Gastric Cancer: Pooled Analysis Study
Source: J Med Internet Res. 2022 May 16;24(5):e27694. doi: 10.2196/27694 (PMC9152716; doi:10.2196/27694)

## Supplementary File 2

### Study quality assessment according to the QUADAS-2

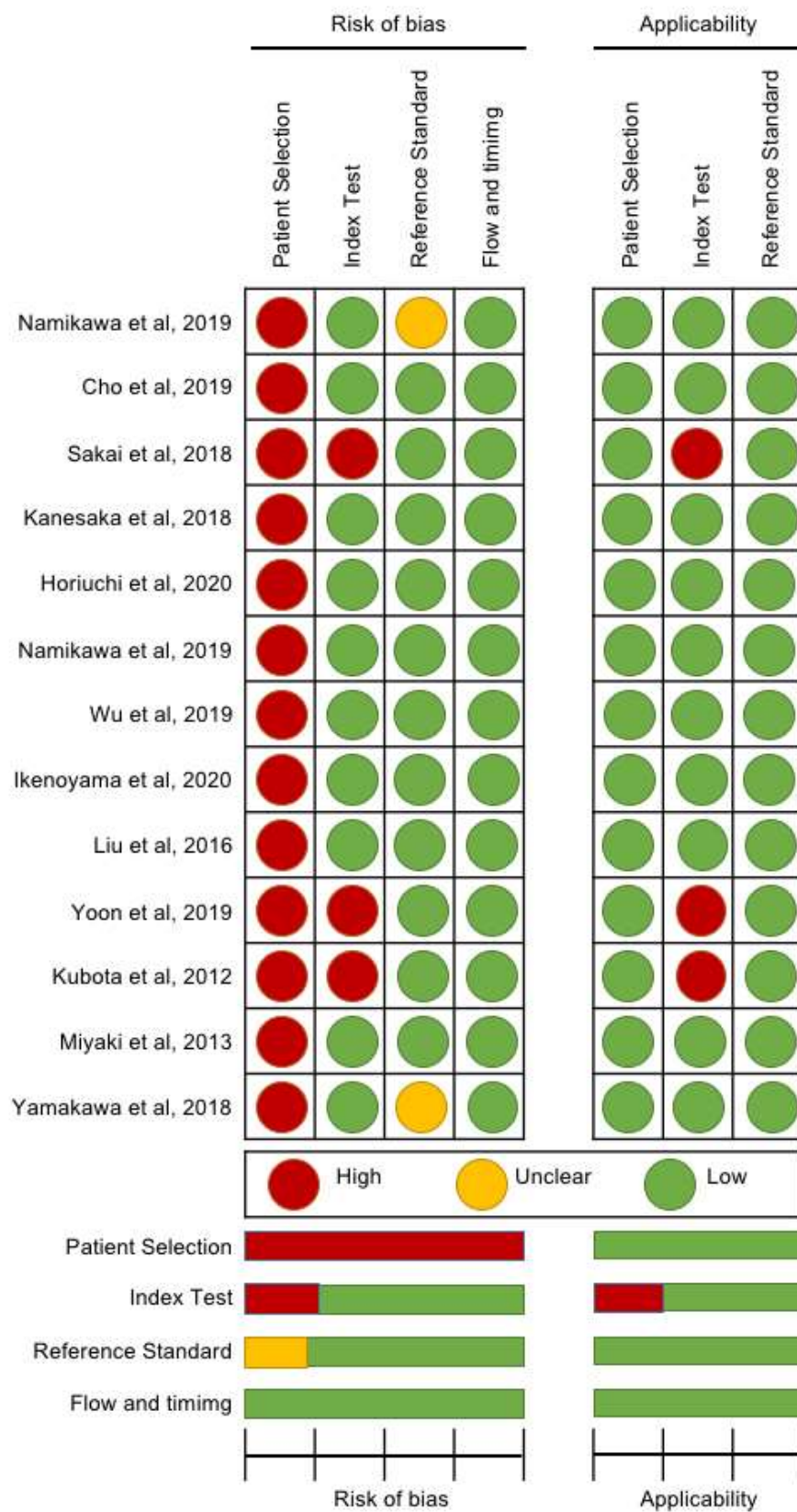

Supplement: Multimedia Appendix 2 [file jmir_v24i5e27694_app2.pdf]

## Supplementary File 4

### Scatter Matrix

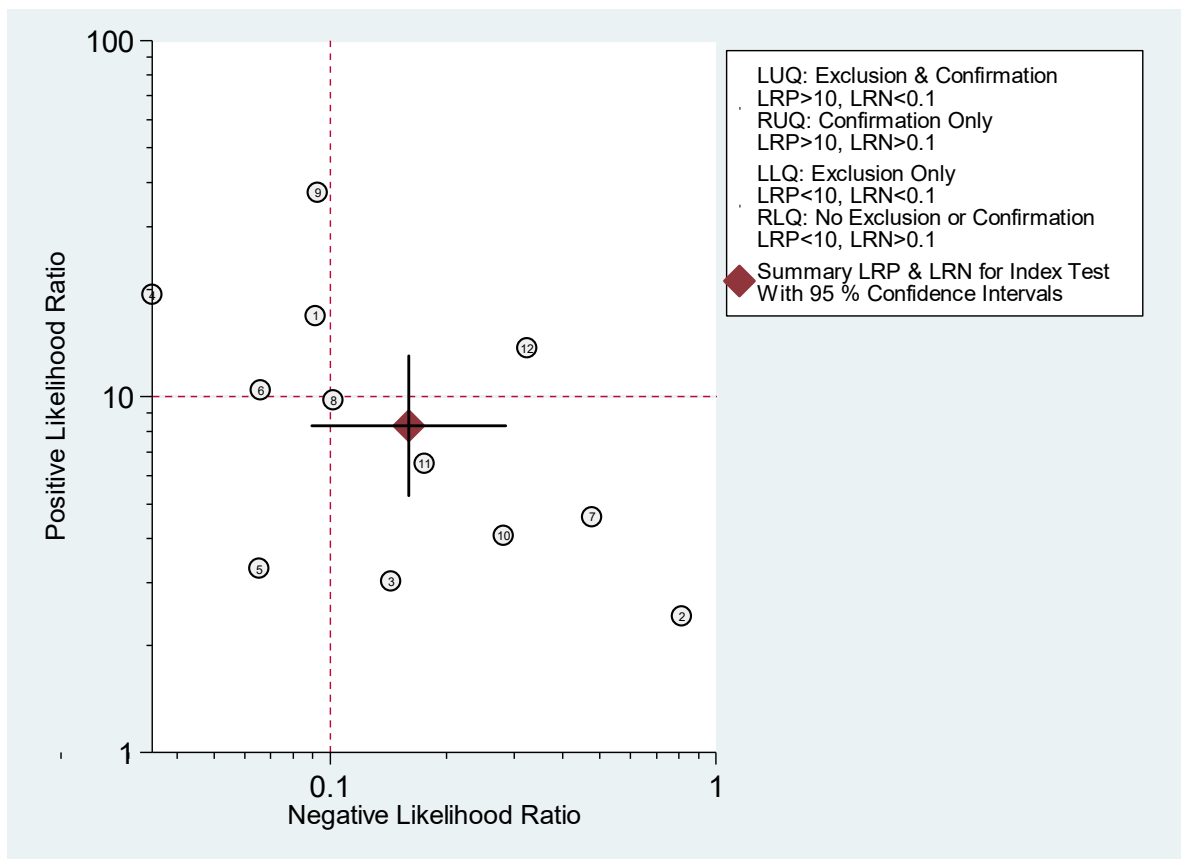

Supplement: Multimedia Appendix 4 [file jmir_v24i5e27694_app4.pdf]

## Supplementary File 5

### Egger's test

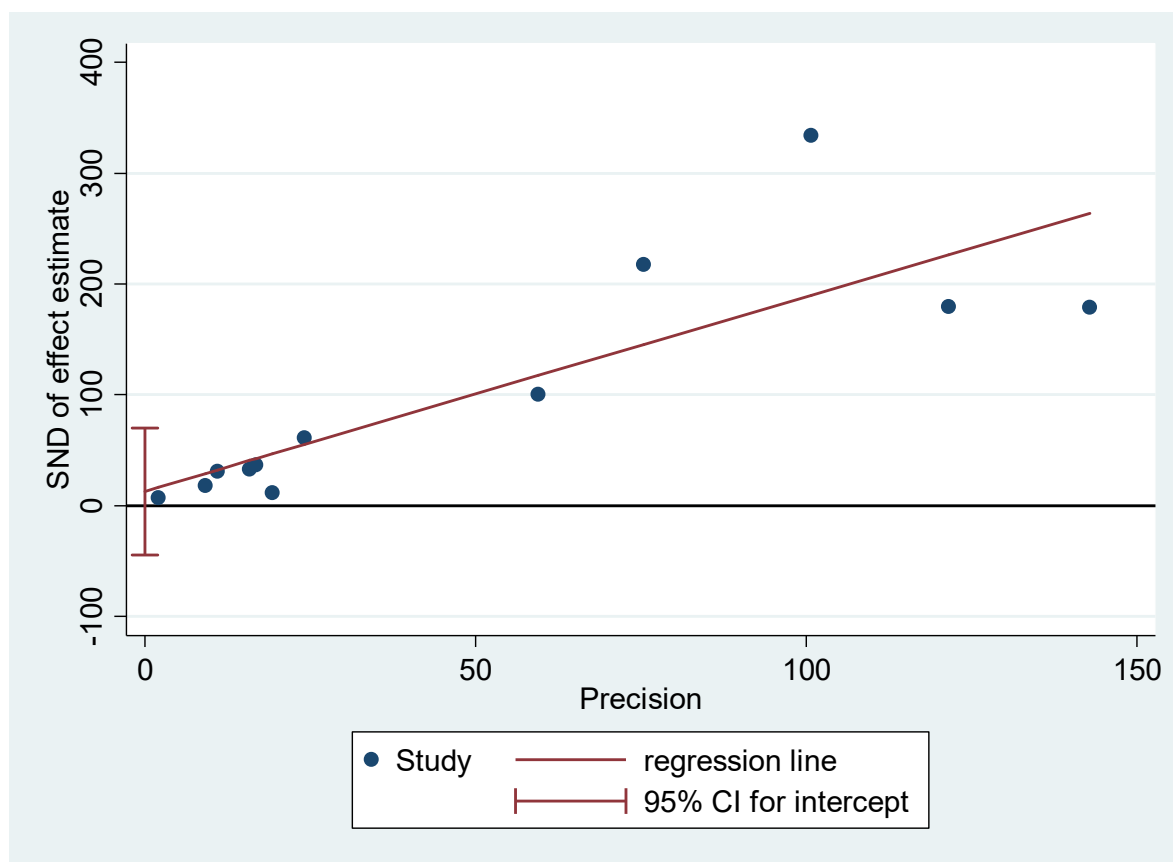

Supplement: Multimedia Appendix 5 [file jmir_v24i5e27694_app5.pdf]

## Supplementary File 6

### Subgroup analysis for studies that used deep learning

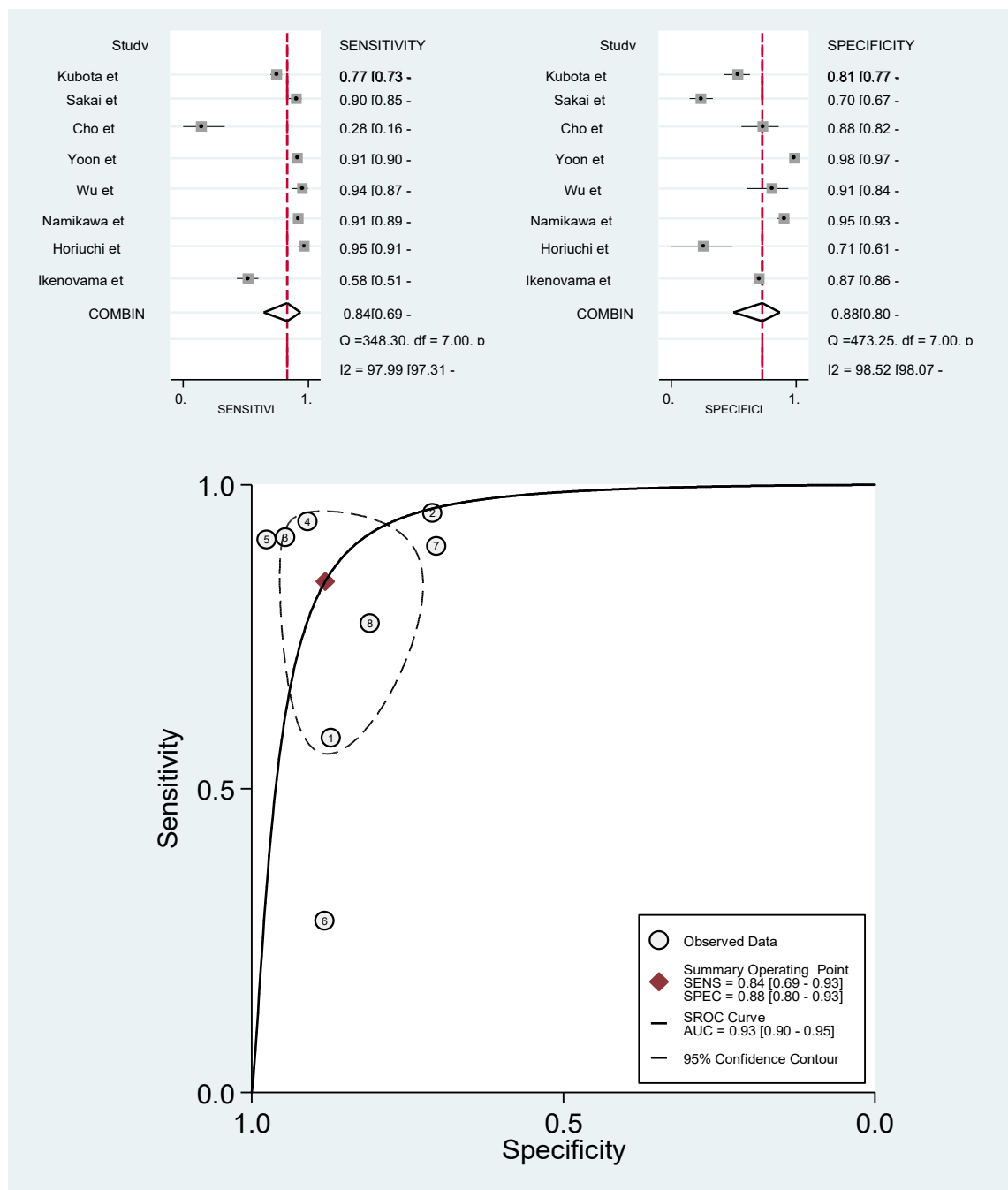

Supplement: Multimedia Appendix 6 [file jmir_v24i5e27694_app6.pdf]

## Supplementary File 8

### Subgroup analysis for studies that used white light image

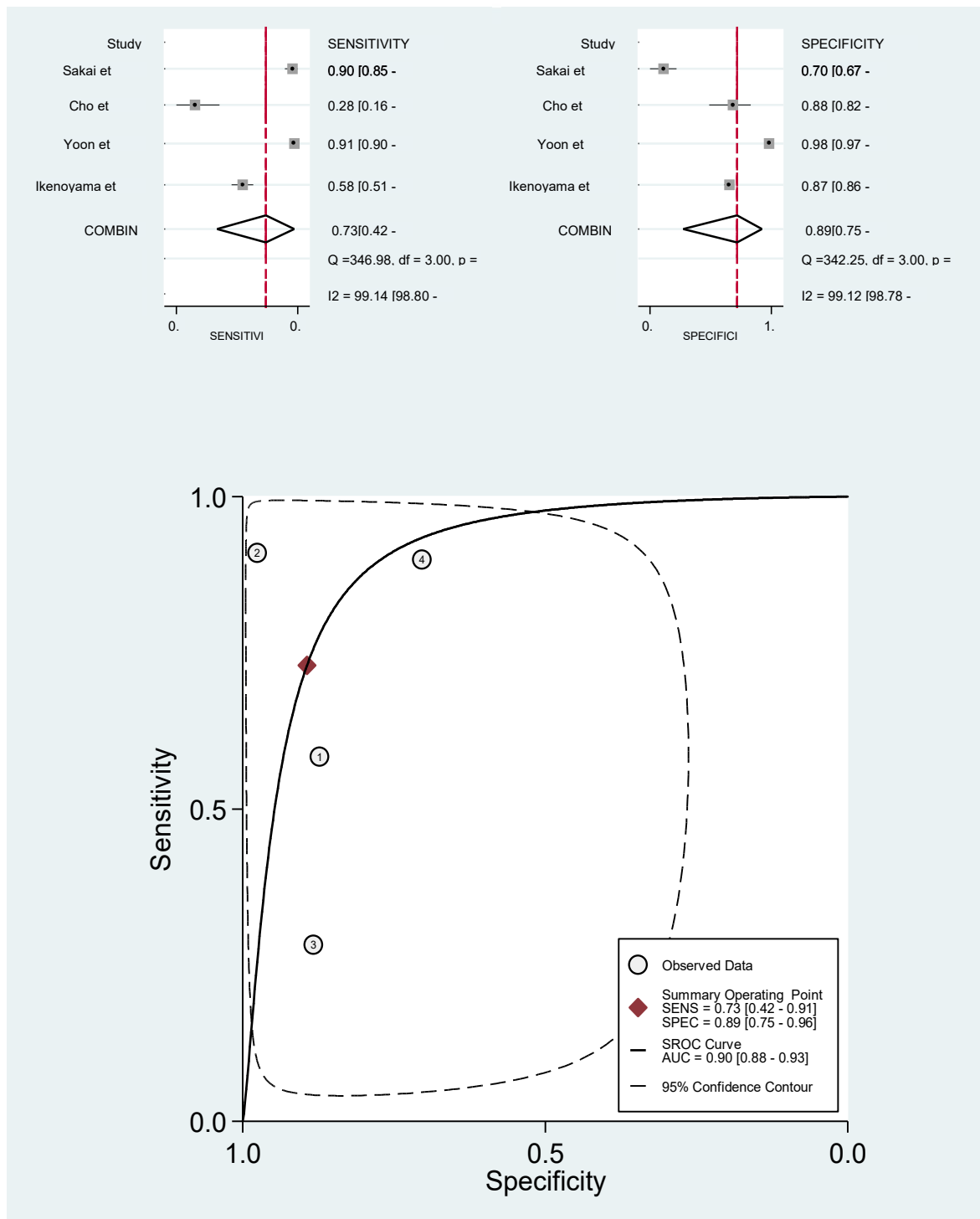

Supplement: Multimedia Appendix 8 [file jmir_v24i5e27694_app8.pdf]

## Supplementary File 9

### Subgroup analysis for studies that used narrow band imaging techniques

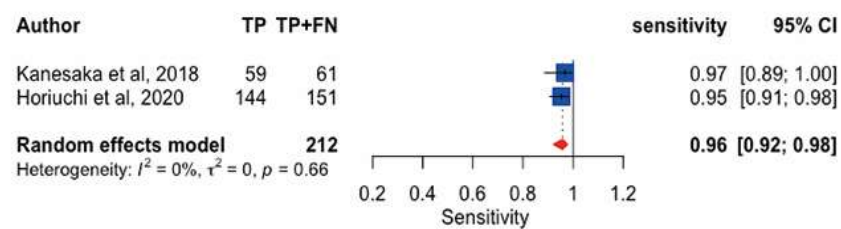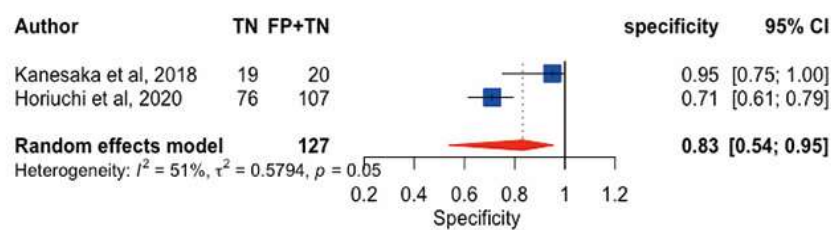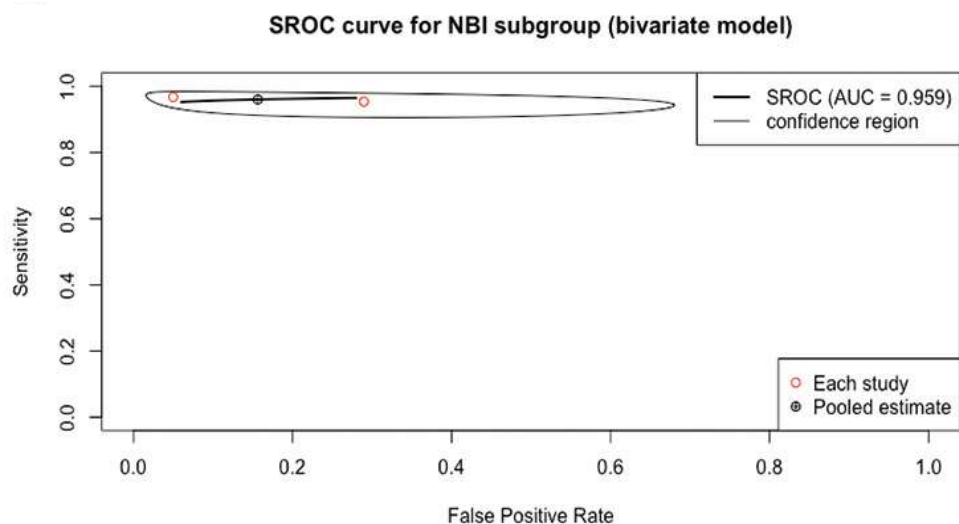

Supplement: Multimedia Appendix 9 [file jmir_v24i5e27694_app9.pdf]
