# Supplementary material for: The Accuracy of Artificial Intelligence in the Endoscopic Diagnosis of Early Gastric Cancer: Pooled Analysis Study
Source: J Med Internet Res. 2022 May 16;24(5):e27694. doi: 10.2196/27694 (PMC9152716; doi:10.2196/27694)

Supplementary File 7
Subgroup analysis for studies without deep learning

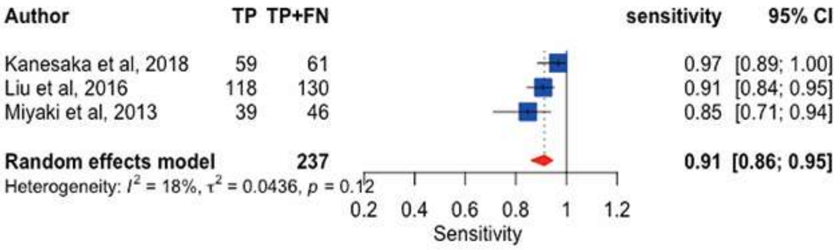

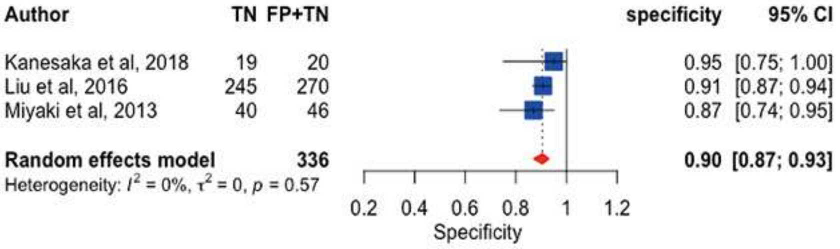

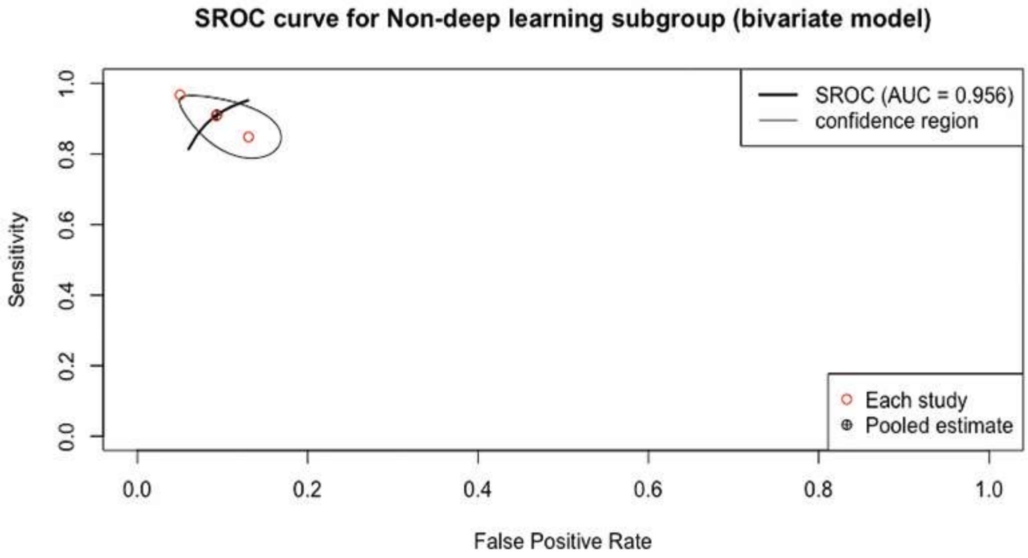

Supplement: Multimedia Appendix 7 [file jmir_v24i5e27694_app7.pdf]
