# Supplementary material for: The Accuracy of Artificial Intelligence in the Endoscopic Diagnosis of Early Gastric Cancer: Pooled Analysis Study
Source: J Med Internet Res. 2022 May 16;24(5):e27694. doi: 10.2196/27694 (PMC9152716; doi:10.2196/27694)
Supplement: Multimedia Appendix 10 [file jmir_v24i5e27694_app10.pdf]

Supplementary Table 1. Characteristics of the studies that compared diagnostic performance of AI to endoscopists and its sensitivity analysis.

Characteristics of the studies that compared diagnostic performance of AI to endoscopist

	Endoscopists				
	Classification	No.	Clinical experience (years)	Endoscopies per year	Image/AI
Cho et al, 2019	N/A	3	6.7	N/A	
Wu et al, 2019	Expert	6	>5	>1000	WLI/CNN
	Senior	8	1-3	>800	Mix/CNN
	Novice	7	<1	50-200	
Ikenoyama et al, 2020	Certified	33	>10	818	
	Uncertified	34	5-10	666	WLI/CNN

The diagnostic performance of AI and endoscopists on early gastric cancer and its sensitivity analysis

Study		Endoscopists			AI		
		Sensitivity	Specificity	Accuracy	Sensitivity	Specificity	Accuracy
Cho et al, 2019	Endoscopists	0.51 [0.43-0.60]	0.90 [0.87-0.93]	0.82	0.28 [0.16-0.43]	0.88 [0.82-0.93]	0.75
Wu et al, 2019	Expert	0.94 [0.79-1.09]	0.87 [0.73-1.02]	0.90	0.94 [0.87-0.98]	0.91 [0.84-0.96]	0.93
	Senior	0.90 [0.78-1.02]	0.85 [0.53-1.17]	0.87			
	Novice	0.75 [0.63-0.88]	0.89 [0.77-1.01]	0.81			
Ikenoyama et al, 2020	Certified	0.37 [0.34-0.41]	0.97 [0.97-0.97]	0.93	0.58 [0.51-0.65]	0.87 [0.86-0.89]	0.85
	Uncertified	0.27 [0.24-0.30]	0.97 [0.97-0.98]	0.92			
All 3 studies		0.68 [0.42-0.94]	0.92 [0.87-0.98]		0.67 [0.26-0.92]	0.87 [0.86-0.89]	-

WLI, white light imaging; CNN, convolutional neural network.
