# Supplementary material for: The Accuracy of Artificial Intelligence in the Endoscopic Diagnosis of Early Gastric Cancer: Pooled Analysis Study
Source: J Med Internet Res. 2022 May 16;24(5):e27694. doi: 10.2196/27694 (PMC9152716; doi:10.2196/27694)
Supplement: Multimedia Appendix 11 [file jmir_v24i5e27694_app11.pdf]

Supplementary Table 2. Sensitivity analysis of the studies that included gastric lesions other than small gastric cancer lesions.

**Excluded studies that only detected small early gastric cancer (< 20 mm)**				
**Study**	**Inclusion criteria**	**Image**	**AI**	**Endoscopist**
Kanesaka et al, 2018	Depressed lesion < 10mm	NBI	SVM	N
Ikenoyama et al, 2020	Early gastric cancer < 20mm	WLI	CNN	Y

**Sensitivity analysis of the remained studies after excluding studies listed above**				
	**Sensitivity**	I^2	**Specificity**	I^2
Remained 10 studies	0.86 [0.75-0.92]	97%	0.89 [0.83-0.94]	96%
**Different AI methods (deep learning and non-deep learning)**				
Deep learning	0.87 [0.72-0.94]	98%	0.88 [0.78-0.94]	97%
Non-deep learning	0.89 [0.84-0.93]	0%	0.90 [0.86-0.93]	0%
**Various imaging modalities (WLI and NBI)**				
WLI	0.77 [0.38-0.95]	98%	0.90 [0.70-0.97]	97%
NBI[a]	-	-	-	-
**Diagnostic performance of AI and endoscopists**				
AI	0.71 [0.15-0.97]	96%	0.89 [0.85-0.93]	0%
Endoscopist	0.77 [0.56-0.98]	93%	0.90 [0.88-0.93]	0%

WLI, white light imaging; NBI, narrow band imaging.
a, study exclusion does not affect previous results.
