# Supplementary material for: The Accuracy of Artificial Intelligence in the Endoscopic Diagnosis of Early Gastric Cancer: Pooled Analysis Study
Source: J Med Internet Res. 2022 May 16;24(5):e27694. doi: 10.2196/27694 (PMC9152716; doi:10.2196/27694)
Supplement: Multimedia Appendix 12 [file jmir_v24i5e27694_app12.pdf]

Supplementary Table 3. Sensitivity analysis of the studies that does not detect early gastric cancer lesions based on pathological grading.

Excluded studies that detected early gastric cancer based on pathological prediction				
**Study**	**Inclusion criteria**	**Image**	**AI**	**Endoscopist**
Cho et al, 2019	-	WLI	CNN	Y
Kubota et al, 2012	-	N/A	Multiple neural network	N

Sensitivity analysis of the remained studies after excluding studies listed above				
	**Sensitivity**	I^2	**Specificity**	I^2
Remained 10 studies	0.89 [0.83-0.93]	94%	0.90 [0.84-0.94]	97%
**Different AI methods (deep learning and non-deep learning)**				
Deep learning	0.90 [0.81-0.95]	97%	0.89 [0.78-0.95]	99%
Non-deep learning[a]	-	-	-	-
**Various imaging modalities (WLI and NBI)**				
WLI	0.83 [0.64-0.93]	98%	0.90 [0.70-0.97]	99%
NBI[a]	-	-	-	-
**Diagnostic performance of AI and endoscopists**				
AI	0.82 [0.45-0.96]	94%	0.87 [0.86-0.89]	0%
Endoscopist	0.72 [0.36-1.09]	98%	0.94 [0.88-1.00]	29%

WLI, white light imaging; NBI, narrow band imaging.
a, study exclusion does not affect previous results.
