# Supplementary material for: The Accuracy of Artificial Intelligence in the Endoscopic Diagnosis of Early Gastric Cancer: Pooled Analysis Study
Source: J Med Internet Res. 2022 May 16;24(5):e27694. doi: 10.2196/27694 (PMC9152716; doi:10.2196/27694)
Supplement: Multimedia Appendix 13 [file jmir_v24i5e27694_app13.pdf]

Supplementary Table 4. Sensitivity analysis of the studies that separated training and testing dataset during AI training.

Excluded studied in subgroup 6 that failed to separate training and testing dataset				
**Study**	**Inclusion criteria**	**Image**	**AI**	**Endoscopist**
Sakai et al, 2018	-	WLI	CNN	N
Yoon et al, 2019	-	WLI	CNN	N
Kubota et al, 2012	-	N/A	Multiple neural network	N

Sensitivity analysis of the remained studies after excluding studies listed above				
	**Sensitivity**	I^2	**Specificity**	I^2
Remained 9 studies	0.85 [0.70-0.93]	96%	0.90 [0.86-0.93]	91%
**Different AI methods (deep learning and non-deep learning)**				
Deep learning	0.82 [0.54-0.95]	98%	0.88 [0.81-0.93]	93%
Non-deep learning[a]	-	-	-	-
**Various imaging modalities (WLI and NBI)**				
WLI	0.44 [0.24-0.66]	85%	0.87 [0.86-0.89]	0%
NBI[a]	-	-	-	-
**Diagnostic performance of AI and endoscopists**				
AI[a]	-	-	-	-
Endoscopist[a]	-	-	-	-

WLI, white light imaging; NBI, narrow band imaging.
a, study exclusion does not affect previous results.
