# Supplementary material for: The Accuracy of Artificial Intelligence in the Endoscopic Diagnosis of Early Gastric Cancer: Pooled Analysis Study
Source: J Med Internet Res. 2022 May 16;24(5):e27694. doi: 10.2196/27694 (PMC9152716; doi:10.2196/27694)
Supplement: Multimedia Appendix 14 [file jmir_v24i5e27694_app14.pdf]

Supplementary Table 5. Sensitivity analysis of the studies with low risk on index test.

Excluded studied that did not mentioned pathological proof of early gastric cancer in text

Study	Inclusion criteria	Image	AI	Endoscopist
Namikawa et al, 2019	-	WLI, NBI, chromo	CNN	N
Yamakawa et al, 2018	-	Not mentioned	Not mentioned	N

Sensitivity analysis of the remained studies after excluding studies listed above

	Sensitivity	I^2	Specificity	I^2
Remained 9 studies	0.86 [0.74-0.93]	98%	0.88 [0.81-0.93]	97%
**Different AI methods (deep learning and non-deep learning)**				
Deep learning	0.83 [0.64-0.93]	98%	0.87 [0.77-0.93]	98%
Non-deep learning[a]	-	-	-	-
**Various imaging modalities (WLI and NBI)**				
WLI	-	-	-	-
NBI[a]	-	-	-	-
**Diagnostic performance of AI and endoscopists**				
AI[a]	-	-	-	-
Endoscopist[a]	-	-	-	-

WLI, white light imaging; NBI, narrow band imaging.
a, study exclusion does not affect previous results.
